# Recurrent Hippocampo-neocortical sleep-state divergence in humans

**DOI:** 10.1073/pnas.2123427119

**Published:** 2022-10-24

**Authors:** Rockelle S. Guthrie, Davide Ciliberti, Emily A. Mankin, Gina R. Poe

**Affiliations:** ^a^Department of Integrative Biology and Physiology, University of California, Los Angeles, CA 90095;; ^b^Molecular Cellular and Integrative Physiology Program, University of California, Los Angeles, CA 90095;; ^c^Department of Neurosurgery, University of California, Los Angeles, CA 90095;; ^d^Department of Psychiatry and Biobehavioral Science, University of California, Los Angeles, CA 90095

**Keywords:** intracranial EEG, scalp EEG, asynchronous sleep, regional sleep, dreaming

## Abstract

Sleep is assumed to be a unitary, global state in humans and most other animals that is coordinated by executive centers in the brain stem, hypothalamus, and basal forebrain. However, the common observation of unihemispheric sleep in birds and marine mammals, as well as the recently discovered nonpathological regional sleep in rodents, calls into question whether the whole human brain might also typically exhibit different states between brain areas at the same time. We analyzed sleep states independently from simultaneously recorded hippocampal depth electrodes and cortical scalp electrodes in eight human subjects who were implanted with depth electrodes for pharmacologically intractable epilepsy evaluation. We found that the neocortex and hippocampus could be in nonsimultaneous states, on average, one-third of the night and that the hippocampus often led in asynchronous state transitions. Nonsimultaneous bout lengths varied from 30 s to over 30 min. These results call into question the conclusions of studies, across phylogeny, that measure only surface cortical state but seek to assess the functions and drivers of sleep states throughout the brain.

## Significance

We report here evidence that human cortical and subcortical brain areas exist in different combinations of simultaneous sleep–wake states throughout the night. These findings contribute to our understanding of the complexity of the mechanisms generating sleep and hold implications for the functions of sleep. Future studies should directly measure or take into consideration the putative sleep state of the region of interest, or risk missing important effects of experimental manipulations on subcortical areas that could be critical to understanding the sleep question at hand.

Sleep has been assumed to be a global behavior that is centrally controlled. The main assumption is that various states occur simultaneously throughout the brain and body, except in abnormal conditions.

The first evidence to suggest the contrary of this widely accepted theory came with the discovery of unihemispheric sleep in birds and seagoing mammals, such as seals, dolphins, and whales (1). Birds can sleep with one eye open, alert to environmental dangers or opportunities, while the brain hemisphere contralateral to the closed eye simultaneously shows all the signs of non rapid eye movement (NREM) sleep (2). Asynchronous sleep onset has been previously described in humans, where increases in sleep spindle density in the hippocampus (3) and slow-wave power in the thalamus (4) occur prior to sleep onset defined using scalp electroencephalography (EEG). In rodents (5–7), nonhuman primates (8), and humans (9, 10), features of different NREM sleep states, such as slow waves and spindles, have been observed within local cortical areas (termed local sleep). In these cases, sleep was scored using cortical electrodes or scalp EEG, while sleep features occurring in local circuits were characterized within this global state.

When objective, standard sleep-scoring criteria were applied independently to signals recorded from the rat hippocampus and parietal neocortex to distinguish NREM sleep from REM, the results yielded different answers to the question “in what sleep state is this animal now?” (11). This study by Emrick et al. (11) found that different simultaneous regional sleep states occurred quite often in transitions between global states. The prevalence of asynchronous sleep states between superficial and deep brain structures in rodents has been observed in other studies as well (12, 13). However, the occurrence and dynamics of different simultaneous sleep states between regions have not been described in humans. The existence of nonsimultaneous sleep states would indicate that sleep-modulating centers of the brain are not as homogeneously functionally connected to their cortical targets as previously hypothesized. More importantly, if deep cortical and subcortical areas frequently exhibit different sleep states from what is assessed in recordings from superficial cortical sites, then studies based on surface cortical sleep alone might miss important manipulation effects on subcortical areas that could be critical to understanding the functions and drivers of sleep.

We report here evidence of different sleep states occurring simultaneously in the human neocortex and hippocampus that fluctuate and reappear regularly throughout the night. These findings encourage caution in interpreting sleep physiology results based solely on surface cortical channels and support the idea of a distributed rather than centralized organization of sleep.

## Results

In this study, we used a publicly available dataset of whole-night EEG from the neocortex central midline (Cz electrode) and intracranial electroencephalography (iEEG) recordings from the posterior hippocampus of eight participants with epilepsy who were implanted with depth electrodes for seizure localization (14). The average recording length was 8.6 ± 1.2 h. Details on participants and how data were collected, including Montreal Neurological Institute (MNI) coordinates, are described in detail in Staresina et al. (15).

**Fig. 1.**
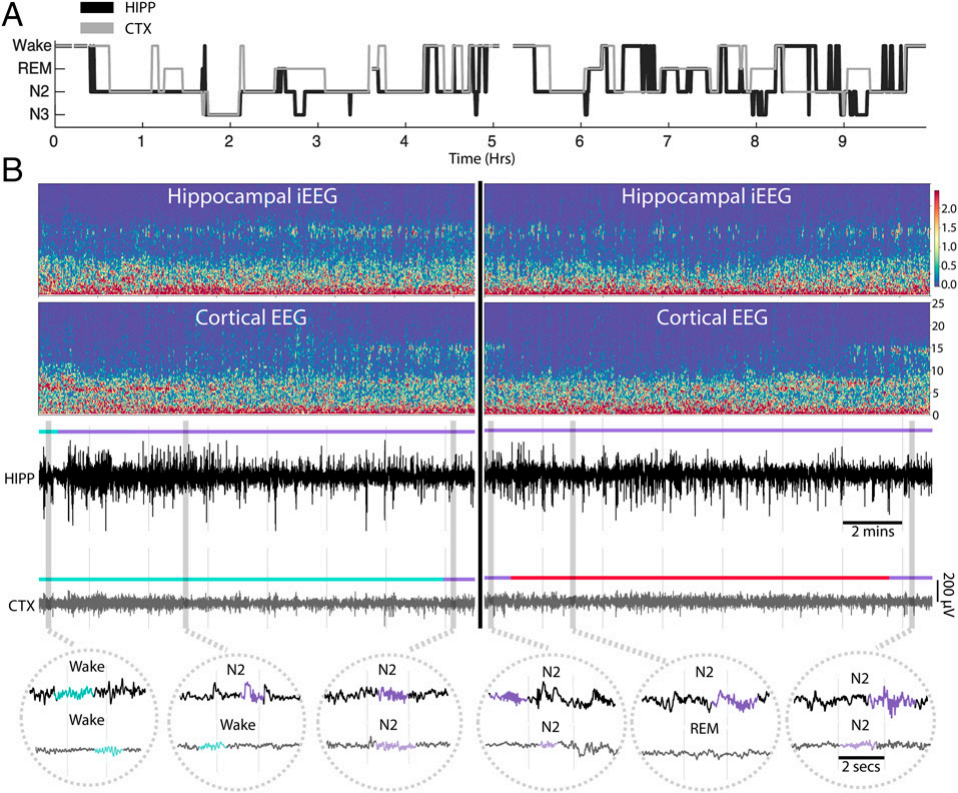
Different sleep states in the human hippocampus and neocortex. (*A*) Hypnogram from a night of sleep with hippocampal iEEG (black trace; HIPP) and neocortical scalp EEG (gray trace; CTX). Note how divergent states between the two brain areas occur not only at sleep onset but also, throughout the night. Epochs overwhelmed with artifact were unmarked and appear as blank spaces. (*B*) Examples of the hippocampus transitioning into N2 sleep before the cortex (*Left*) and the cortex going into REM independently of the hippocampus (*Right*). *Upper* shows normalized spectrograms for 15-min windows from the hypnogram in *A*. In *Lower*, bandpass filtered (0.1 to 30 Hz) signals for the hippocampus and cortex are shown. Turquoise, wake; purple, N2; red, REM. In *Insets*, signal traces with instances of theta, spindles, and/or K complexes (turquoise, theta; purple, spindles and/or K complexes) used for scoring are shown. Hippocampal and cortical independent state assignments are listed above the corresponding trace.

We sought to answer the question of whether the human hippocampus and cortex exhibit asynchronous state transitions beyond sleep onset and throughout the course of the night. Each recording in the hippocampus and cortex was scored independently by an expert reviewer using standard scoring criteria (16) at each site (*Materials and Methods*). Nonsimultaneous states were present throughout the night among all subjects, and the amount of time spent in each state was region specific. The hypnogram from a single subject in Fig. 1*A* shows asynchronous state transitions between the hippocampus and cortex throughout the night. An example of the hippocampus transitioning into NREM stage 2 (N2) sleep prior to the cortex can be seen in Fig. 1 *B*, *Left*. In this example, both the hippocampus and cortex began in waking simultaneously, but with the occurrence of local sleep spindles and K complexes, the hippocampus transitioned into N2 sleep about 14 min prior to the cortex, which remained in waking. Fig. 1 *B*, *Right* shows the cortex transitioning into and out of REM sleep independent of the hippocampus, which remained in N2 sleep for the entire cortical REM bout. We found independent sleep states occurring between the cortex and hippocampus throughout sleep in all subjects studied (*SI Appendix*, Fig. 1).

### Nonsimultaneous States Are Prevalent throughout the Night.

**Fig. 2.**
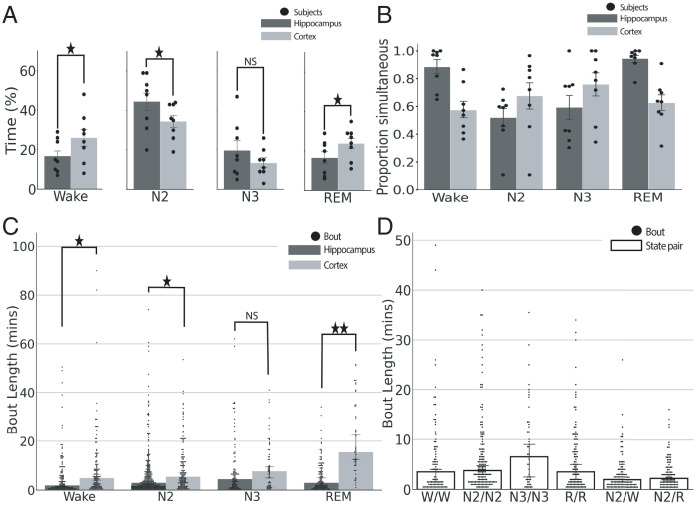
Nonsimultaneous states between the hippocampus and cortex are prevalent throughout the night. (*A*) Percentage of time in each state for the hippocampus and cortex (*n* = 8 subjects). ^★^*P* < 0.05; NS = nonsignificant. (*B*) Proportion of epochs in simultaneous state pairs for the hippocampus (dark gray) and cortex (light gray; *n* = 8 subjects). (*C*) Median bout length for the hippocampus and cortex. ^★^*P* < 0.05; ^★★^*P* < 0.0001; NS = nonsignificant. (*D*) Median bout length for state pairs. The hippocampal state is listed first, and the cortical state is listed second (e.g., N2/W is hippocampus in N2 and cortex in wake). For *A* and *B*, bar height indicates the mean across subjects, error bars represent the SEM, and dots indicate the average for a single subject. In *C* and *D*, dots indicate the duration in minutes of each bout. Bar height shows the median, and error bars indicate 95% CIs. The number of bouts are as follows: hippocampal wake = 121, cortical wake = 118, hippocampal N2 = 267, cortical N2 = 135, hippocampal N3 = 89, cortical N3 = 56, hippocampal REM = 115, cortical REM = 47. W/W = 95, N2/N2 = 156, N3/N3 = 49, R/R =107, N2/W = 124, and N2/R = 98 (*SI Appendix*, Table 1). W = wake; R = REM.

To determine the amount of time the hippocampus and cortex spent in each sleep–wake state, we calculated the percentage of time spent in waking, N2, NREM stage 3 (N3), and REM sleep separately for each region. We found that the cortex spent a greater amount of time in waking and REM than the hippocampus, while the hippocampus spent a greater amount of time in N2 sleep. There were no significant differences between regions for N3 sleep (Fig. 2*A*) (mean ± SEM: wake: hippocampus: 15 ± 3%, cortex: 24 ± 4%, *P* = 0.0088; REM: hippocampus: 17 ± 3%, cortex: 25 ± 3%, *P* = 0.0047; N2: hippocampus: 46 ± 5%, cortex: 36 ± 3%, *P* = 0.047; N3: hippocampus: 21 ± 6%, cortex: 15 ± 3%, *P* = 0.26, two-sided paired *t* test).

Next, we asked how common it was for the two regions to be in nonsimultaneous sleep states. On average, participants spent about one-third of the night in asynchronous sleep states (mean ± SEM: 33.92 ± 2.9%; range: 25.3 to 49.6%) (*SI Appendix*, Fig. 1*B*).

To test whether the probability of being in a simultaneous state was dependent on the state or region, for each region we computed the proportion of time that the other region was in the same state by expressing the number of epochs of simultaneous states as a fraction of total time spent in that state within the respective region. We found that the proportion of simultaneous states varied both by state and by region (Fig. 2*B*). When waking or REM appeared in the hippocampus, the same state was often also found in the cortex (wake: 88%, REM: 94%). However, when N2 or N3 sleep was present in the hippocampus, these NREM states were commonly not found in the cortex (N2: 52%, N3: 59%). The cortex showed a different pattern than the hippocampus. Waking and REM states in the cortex would more often not be simultaneously identified in the hippocampus. That is, 57% cortical waking was not found simultaneously in the hippocampus, and 62% of cortical REM was not simultaneously found in the hippocampus. Conversely, NREM stages of sleep scored in the cortex shared a greater state commonality with the hippocampus; cortical N2 occurred simultaneously with hippocampal N2 67% of the time, and cortical N3 shared the state with the hippocampus 76% of the time.

### Bout Lengths Vary by Region and State.

We sought to characterize the amount of time spent in single bouts of each sleep state within the hippocampus and cortex independently. We first computed bout lengths within each region without regard to the state of the other region. We found that the cortex had longer bout lengths than the hippocampus for waking, N2, and REM (wake *P* = 0.0485, N2 *P* = 0.012, REM *P* < 0.0001, linear mixed effects model with maximum likelihood estimation with participant random intercept and region fixed effects). Bout lengths for N3 were not significantly different between areas (Fig. 2*C*) (*P* = 0.596).

We next compared the duration of bouts (bout lengths) for simultaneous and nonsimultaneous states. The median bout length was longer for synchronous than for asynchronous state pairs. Median bout length in minutes for synchronous states were wake/wake: 3.5, REM/REM: 3.5, N2/N2: 3.75, and N3/N3: 6.5. All nonsimultaneous state pairs had medians less than 2.5 min (Fig. 2*D*). However, there were long upper-length tails in both sets of distributions. Surprisingly, the nonsimultaneous states could last for quite a long time, with maximum lengths of N2/N3: 35 min, N3/N2: 31 min, N2/wake: 26 min, N2/REM: 16 min, and REM/N2: 10 min (*SI Appendix*, Table 1).

### Variability in Observed State Pair Combinations.

Although nonsimultaneous wake, N2, N3, and REM sleep were readily observed across all subjects, we noticed that some state pair combinations were much more common than others. Some state pairs were only seen in a subset of participants. For example, only three subjects exhibited the state pair combination of the hippocampus scored in REM, while the cortex was simultaneously scored as being in N2 sleep. Epochs where the hippocampus was scored as N3 and the cortex was simultaneously scored as being in REM were observed in four subjects (*SI Appendix*, Table 1). Bout durations for REM/N2 and N3/REM could last up to 10 and 7.5 min, respectively (median length REM/N2 = 1 min, N3/REM = 1.5 min). Some state pair combinations were never observed, including wake/REM, REM/wake, and wake/N3.

**Fig. 3.**
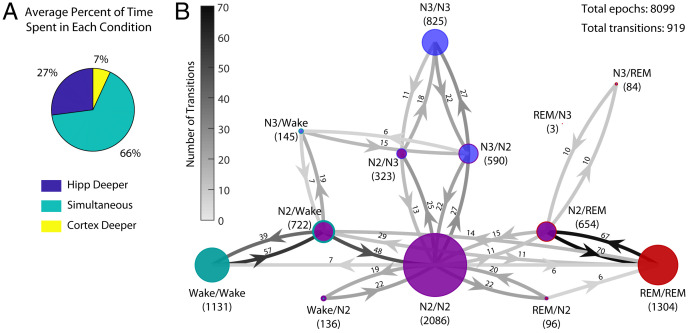
The hippocampus leads transitions into deeper stages of sleep. (*A*) The fraction of time one region is in deeper sleep than the other. When not in simultaneous sleep, the hippocampus is much more often in deeper sleep than the cortex. This figure shows the mean values across participants. Individual participant data are in *SI Appendix*, Fig. 1. (*B*) A directed graph showing how state pairs transition into each other. Each node represents one possible state pair; the labels show the state pair and the number of epochs (summed across all participants) that were scored as that pair. The size of the nodes reflects the relative prevalence of each state pair, and colors represent the states, with the inner color showing the state of the hippocampus (wake, teal; N2, purple; N3, blue; REM, red) and the outer color showing the state of the cortex (very small nodes show only the hippocampal color). The directed edges of the graph show the total number of times a transition between two state pairs occurred. To simplify the graph, edges with five or fewer transitions are not shown. Note that the total number of transitions is much smaller than the total number of epochs because long bouts of a single state pair account for many epochs but only one transition to/from that bout.

In general, we found that during nonsimultaneous state periods, the hippocampus was more often in a deeper sleep state than the cortex and preceded the cortex into deeper sleep stages (Fig. 3). Only one participant showed a higher prevalence for the cortex to be in a deeper state than the hippocampus, and in two participants, the amount of time in which the cortex was in deeper sleep than the hippocampus was less than 1% (*SI Appendix*, Fig. 2).

### State Transition Patterns.

Finally, we sought to fully characterize the dynamics of how the state pairs transition during the night. For each possible pair of states, we calculated the fraction of total transitions that occurred between each pair. We found that some transitions were much more common than others, such that the sleep architecture could be described with N2/N2 as a major hub and the other simultaneous sleep states as spokes radiating off the hub. The intermediate nonsimultaneous state pairs (e.g., N2/wake and N3/N2) serve as way stations between the simultaneous pairs (Fig. 3). Although other transitions were possible, they were less common. Interestingly, we did not observe transitions directly from one simultaneous sleep state to another—one region always led the way. Further research that explores the mechanisms that give rise to this sleep architecture would be valuable.

### Sleep–Wake State Determination for the Hippocampus and Cortex Follows the Standard State Scoring Criteria.

**Fig. 4.**
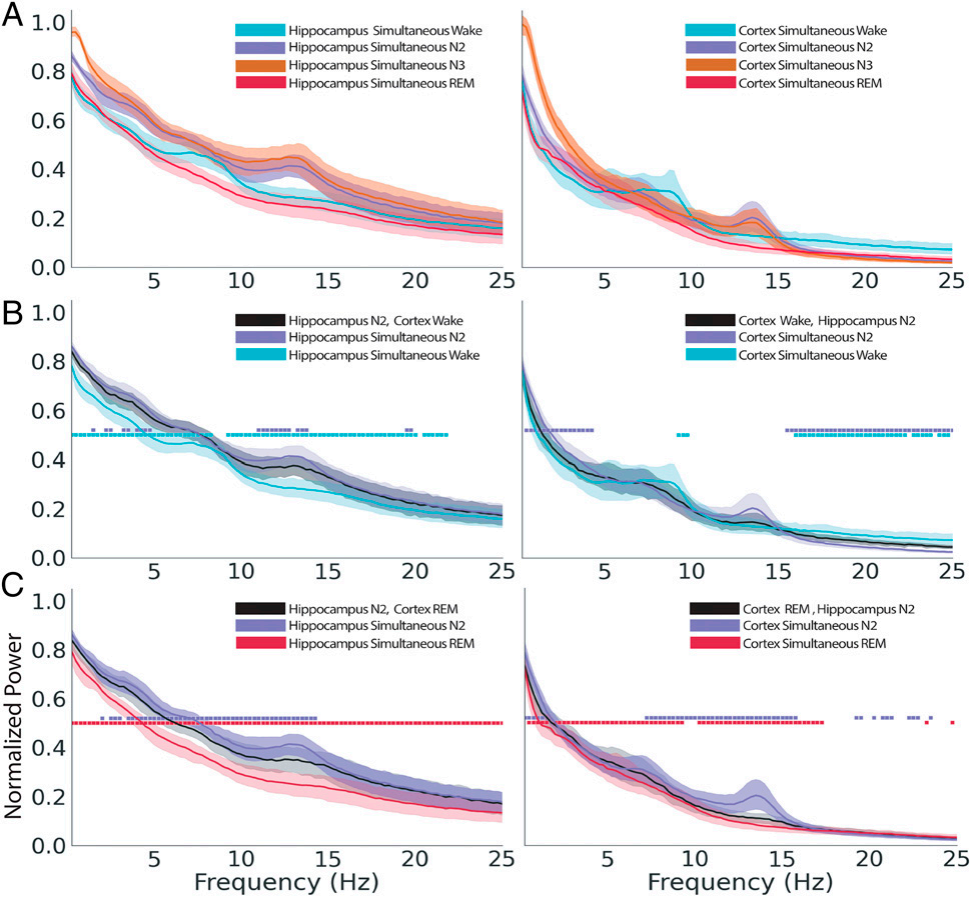
PSD profiles show typical variation across sleep and waking states, and the PSD resembles the locally scored state during asynchronous periods. (*A*) Mean PSD across eight subjects for simultaneous states in the hippocampus (*Left*) and cortex (*Right*). (*B*) Mean PSD across eight subjects for the hippocampus (*Left*) and cortex (*Right*) for all epochs where the hippocampus was scored as N2 and the cortex was simultaneously scored as being in waking. (*C*) Mean PSD across eight subjects for epochs where the hippocampus (*Left*) was scored as N2 and the cortex was scored simultaneously as REM (*Right*). In *B* and *C*, mean normalized PSD of the nonsimultaneous state is shown with the mean PSD of the corresponding simultaneous state and the mean PSD for the simultaneous state scored at the other site. All PSDs are expressed as a percentage of the sum of power across all simultaneous states and frequencies (0.25 to 30 Hz). Shaded regions correspond to the 95% bootstrap CIs for each frequency bin. Squares indicate significant differences at each frequency bin between the mean PSD of the nonsimultaneous state and the corresponding simultaneous state (▪ indicates *P* < 0.05).

To confirm that the presence of nonsimultaneous sleep states did not arise from spurious issues with our manual sleep scoring, we evaluated the power spectral density (PSD) profiles from each recording within each state as scored in order to test whether scoring in each site matched standard criteria. Sleep scoring for each region was performed separately, and each site showed standard patterns of EEG power for each state as originally defined by the sleep-scoring manual of Rechtshaffen and Kales (16) (Fig. 4). We first evaluated the PSD for periods when the hippocampus and cortex were scored as being in the same state (Fig. 4*A*). As expected, slow-wave sleep (N3) was dominated by high-amplitude slow fluctuations in voltage, whereas the intermediate N2 stage of sleep was dominated by spindles with or without the presence of K complexes in both regions. In both regions, the waking stage was characterized by low power in the slow-wave range (0.25 to 4 Hz) and an increase in power in the theta (4 to 10 Hz) range. The N2 stage was characterized by greater power in the slow-wave range and in the spindle band (11 to 15 Hz), and recording sites showed the greatest amount of power in the slow-wave range during N3 sleep. An increase in power relative to waking and REM sleep in the spindle band was also a characteristic of N3 sleep in both regions. REM sleep in both areas was characterized by low power in the slow-wave and spindle frequency bands and lower power in the 7 to 10 Hz frequency range compared with waking.

### PSD Profiles Are Consistent between Simultaneous and Nonsimultaneous Epochs.

We next explored whether the distribution of power in nonsimultaneous state pairs was similar to that of homogeneous state pairs for each site across the frequency range of 0.25 to 30 Hz commonly used to score sleep. We hypothesized that the PSD profile for the state scored in the region of interest during a nonsimultaneous state pair would match that of the same region’s mean PSD for the same scored state during a simultaneous state. For example, if the hippocampus was scored as being in N2 while the cortex was simultaneously scored as being in REM sleep, the hippocampal signal on average was expected to be more similar to the hippocampal frequency composition when N2 was scored in both sites simultaneously compared with when scored as simultaneously in REM sleep. Specifically, we compared the mean PSD for all epochs of simultaneous N2 with epochs where one region was scored as N2 and the other was scored as wake or REM (Fig. 4 *B* and *C*).

In Fig. 4 *B*, *Left*, we show the PSDs of the hippocampus when scored as N2 sleep while the cortex was simultaneously scored as being in wake (N2/wake) and compare it with simultaneous N2 and simultaneous wake. In this state pair, the hippocampus shows more frequencies significantly different from the simultaneous waking state than the simultaneous N2 state. That is, the hippocampus PSD reflects the true N2 state, despite the cortex being in waking. At the same time, when the cortex is in the N2/wake state (Fig. 4 *B*, *Right*), the cortical PSD more closely reflects the simultaneous waking state, particularly in the slow-wave frequencies that normally dominate in sleep.

When the hippocampus is in N2 and the cortex is in REM (N2/REM) (Fig. 4*C*), the hippocampal PSD differs from REM at every frequency, underscoring its true N2 state. However, the hippocampus in N2/REM shows lower power than simultaneous N2 at most frequencies from 2 to 14.5 Hz. Interestingly, despite being scored as REM, the cortical PSD in N2/REM is also significantly different from simultaneous REM for most frequencies between 0.75 and 17.25 Hz.

Five of the eight subjects also showed N2 in the hippocampus while the cortex was in N3, while seven showed the inverse, N3/N2 (*SI Appendix*, Table 1). However, the main distinction between these non-REM sleep states is the relative amount of slow waves (greater in N3) and spindles (greater in N2). Thus, we did not further characterize their nonsimultaneity.

In general, the distribution of frequencies within each individual region more closely matches the respective scored state when compared with the state scored at the alternate site. In the few cases where the frequencies show significant differences, it appears that the nonsimultaneous power is intermediate between its own state and the alternate state, although always closer to the power in the simultaneous state.

## Discussion

We found that the human hippocampus is often in a different state than the neocortex throughout the night, replicating our previous discovery across sleep in rodents (11). These common nonsimultaneous sleep states occupied approximately one-third of the night. A bout of asynchronous states could last for as long as 35 min, and the hippocampus often led the state transition, while the neocortex followed. The PSD of each region during the nonsimultaneous state resembled that of the same state during simultaneous sleep in the slow-wave, theta, sigma, and beta ranges, with exceptions of intermediate power in select frequency bands in some nonsimultaneous epochs.

The finding that different simultaneous sleep states commonly occur in brain areas throughout the night indicates that explorations of sleep functions need to take into account sleep spatiotemporal specificity. The sleep state determined with a neocortical electrode cannot be assumed to indicate the state of subcortical structures. Studies examining the effects of sleep on physiological and cognitive functions should be viewed in light of the fact that the hippocampus can be in a sleep state not indicated by surface cortical signals.

### Modularity in Sleep-Generating Structures.

It has been commonly thought that executive sleep-inducing brain structures project homogeneously throughout the forebrain. However, recent studies have revealed anatomical modularity in the projection patterns of areas strongly influential to sleep, such as the basal forebrain (17) and locus coeruleus (18–20). That is, subsets of cells in the locus coeruleus work together as a module in the prefrontal cortex, whereas other subsets work in the motor cortex and still others in the hippocampus. These individual modules can act independently of one another and could promote arousal or sleep at different times to different regions. We found that some nonsimultaneous state pairs were common, being observed across all subjects, while others were only seen in a few patients, and some were never observed at all, such as wake/REM and wake/N3. The lack of some state pair occurrences may indicate a limit to the modularity of sleep-state production in the thalamus, hypothalamus, and brain stem. Given the regional modularity of these widely projecting sleep–arousal nuclei, it should not be not entirely surprising that sleep itself is also regionally modular.

### The Hippocampus Leads State Pair Progressions.

Among the common nonsimultaneous state pairs, we found that the hippocampus often entered sleep from waking prior to the cortex as determined by the presence of sleep spindles (Fig. 3), lending support to prior rodent work in our laboratory and others showing an asynchronous onset of sleep (11, 12). Replicating a prior report in humans at sleep onset (3), we found that humans can have an “offline” sleeping hippocampus during cortical waking. Sarasso et al. (3) found that hippocampal spindle density increased in the 30-min window prior to sleep onset in humans and that spindle characteristics were not significantly different between those detected during NREM sleep and those detected prior to sleep onset. These findings lend support to our own definition of sleep onset for both the hippocampus and neocortex using the same criteria—appearance of sleep spindles alone or coupled with K complexes—and scoring the onset of sleep independently for both regions.

Sleep spindles are of thalamic origin and are commonly used in determining sleep onset in cortical areas (16). Increases in sleep spindle density around sleep onset vary by electrode site, with the hippocampus showing the earliest increase (3). The nonsimultaneous increase in sleep spindles across brain structures at sleep onset could reflect modularity in thalamic outputs to these areas, allowing heterogeneity in the timing of their inputs to different cortical structures. For example, chemogenetic inhibition of cells in sensory areas of the thalamic reticular nucleus caused a reduction in observed sleep spindles specifically in the corresponding somatosensory cortex (21). Taken together, these results reveal that nuclei-generating spindles and slow oscillations in the modular thalamus may play a role in the asynchronous appearance of these sleep features at sleep onset.

We also found that, in addition to leading the transition into N2 sleep from wakefulness, the hippocampus is also more likely to transition into N3 and REM sleep prior to the cortex. As nonsimultaneous sleep states were prevalent throughout the entire length of the recording, it stands to reason that there may also be region-dependent modularity in the activity of various N3 and REM sleep–promoting areas, such as the basal forebrain, regions of the hypothalamus, and various brain stem nuclei, throughout the night. Our analyses further develop prior human sleep-onset findings by scoring the entire length of the night’s sleep independently for the hippocampus and cortex, allowing the comparison of sleep-stage dynamics and sleep feature properties in nonsimultaneous states beyond the sleep-onset window. Overall, across the sleep period, nonsimultaneous state transitions were more common than simultaneous transitions, replicating observations in rodents (11, 12).

In instances where the hippocampus was scored as N2 and the cortex was scored as either wake or REM, the frequency composition of the cortical signal was intermediate between that found in N2 sleep and wake or REM. The scalp electrode on the cortex gathers signals from a broader area of tissue than the intracranial electrode and could be registering some slow waves and spindles from distant cortical or subcortical areas (6, 7). Alternatively, the nonsimultaneous state with intermediate cortical PSDs could indicate a unique local neocortical state. Future studies are needed to explore the simultaneity of sleep states among and between neocortical areas.

### Evidence of Nonsimultaneous States between Cortical Areas.

The analyses presented herein were limited to the channels provided through the Open Science Framework (14). However, previous work using multisite sleep scoring provides some evidence of nonsimultaneous sleep among cortical electrodes (7, 12) in rodents. Durán et al. (12) scored the sleep of adult rats from prefrontal, frontal, and parietal cortical signals independently of one another. They reported differences in the amount of time spent in wake, intermediate (N2) sleep, and REM sleep between cortical areas. Nonsimultaneous transitions between sleep stages were common among the cortical areas as well.

Soltani et al. (7) subsequently analyzed multiple cortical recording sites spanning the anterior–posterior axis of the cortex in young and aged mice. They used an automatic sleep-scoring algorithm to determine sleep stages for each individual channel. They replicate the prior report of Durán et al. (12), showing that the amount of time spent in each state (wake, slow-wave sleep, and REM) varied by cortical area in adult mice. They found that asynchronous state transitions were frequent even between neighboring regions. This rodent work suggests that it is possible that different simultaneous states are just as prevalent between human neocortical areas as what we report here between the hippocampus and neocortex.

Although the present study was conducted in participants with epilepsy, it is likely that these findings also apply to healthy humans. Pathological activity throughout sleep in these subjects was limited to epileptic activity, and they showed no abnormal dissociated states, such as REM behavioral disorder, sleep walking/talking, night terrors, or confusional arousals (22). Follow-up studies using magnetoencephalography (MEG) in healthy humans could also help elucidate how often nonsimultaneous sleep states occur at different sites.

### A Role for Nonsimultaneous States in Learning and Memory.

The function of sleep for learning and memory is likely closely linked not only to which sleep stages are modulated following learning but also, to where these changes occur. Prior work has shown that the engagement of task-relevant brain areas during waking induces a local increase in EEG slow-wave power during sleep when compared with adjacent brain areas in rodents (5, 23) and humans (24, 25). Future research is needed to determine whether or how learning influences the simultaneity of states between regions. It may be that some of the reports showing no functional role for sleep for learning could be due to the placement of electrodes outside of the target region of interest when assessing and manipulating sleep states. The benefit of sleep may be dependent on the specific brain areas mediating the behavior (26, 27). If, for example, in a hippocampus-dependent task, REM sleep is deprived based on the appearance of cortical REM without regard to the hippocampal state, then functionally relevant REM could occur in the hippocampus and accomplish memory consolidation. In addition, the brain areas involved in functions of sleep may change across sleep cycles. For example, Ribeiro et al. (28) showed that the areas undergoing plasticity during sleep moved from those proximal to the hippocampus to secondary and tertiary cortical association areas across progressive cycles of sleep. In studies across different learning modalities, sleep spindles and intermediate N2 sleep both increase after learning and predict acquisition (29). Future studies should examine whether such changes are uniform between cortical and subcortical structures.

Another possibility is that nonsimultaneous states could themselves be critical to memory consolidation. Specifically, different neurotransmitter levels are required to produce certain memory-linked sleep signatures, like slow oscillations, theta, and sleep spindles. Nonsimultaneous sleep states could facilitate the coincidence of these differing signatures to accomplish unique functions for learning and memory. For example, the prevalence of K-complex, spindle-rich N2 sleep in the hippocampus at the same time that desynchronized, theta-rich REM sleep is present in the cortex could signal a unique opportunity for memory consolidation. The release of plasticity-inducing acetylcholine and loss of synapse-strengthening norepinephrine during cortical REM while the hippocampus remains in N2 sleep could allow the neocortex to revise memories under the guidance of strong memory replay from the hippocampus, when the local hippocampal neurochemical environment promotes preservation of memories. The question of whether synapses are homeostatically regulated and other possible functions of sleep depend on the accurate measurement of sleep states within the region of interest.

### Implications of Nonsimultaneous REM Sleep on Dreaming.

Reports of perceptually and emotionally vivid dreams occurring during NREM sleep led to the development of the model of covert REM sleep (30, 31). While scoring sleep using traditional methods, some epochs of REM sleep may be “missed” due to a lack of meeting all standard feature characteristics. Most features of REM sleep may be present, but a lack of eye movements or concordant reduction in muscle tone leads the scorer to mark such epochs as NREM stage 1 (N1) or N2 sleep. Thus, an episode of covert REM is defined as “any episode of NREM sleep for which some REM sleep processes are present, but for which REM sleep cannot be scored with standard criteria” (30, 31). Using electrophysiological data from the hippocampus and cortex, we found that epochs of REM sleep can occur within the hippocampus independently of the cortex, lending support to the idea of covert REM. It is thought that these covert REM episodes, which cannot not be readily observed when relying solely on scalp electrodes, give rise to the vivid dreaming that is sometimes reported following NREM sleep awakenings in healthy subjects.

Our findings might also help decipher the phenomenon of lucid dreams. Lucid dreams are rare and known to occur predominantly during REM sleep, where dreams are most vivid. A lucid dream is distinct from typical dreaming due to the awareness of the dreamer that they are in a dream (32, 33). Recent systematic investigations into REM sleep lucid dreaming have explored “interactive dreaming,” in which investigators engage in two-way communication with trained lucid dreamers. Dreamers were trained to respond to external cues through volitional gaze changes or facial or finger movements while maintaining the dreaming state (34). Although investigators were able to successfully communicate with subjects while lucid dreaming, these attempts were unsuccessful ∼60% of the time. We found that across subjects, the cortex is in REM sleep simultaneously with the hippocampus ∼60% of the time (Fig. 2*B*). It may be that in order for the dreamer to become aware that they are dreaming, the hippocampus and cortex must be in asynchronous states. Further research is needed to determine how and through which mechanisms nonsimultaneous sleep stages between brain areas influence vivid and lucid dreams.

### Implications of Nonsimultaneous Sleep States on Sleep Disorders.

There are a number of disorders characterized by what has been traditionally interpreted as sleep-state misperception (SSM) on the part of the patient. SSM is defined as occurring when objective actigraphy or polysomnography contrasts with subjective sleep estimates (sleep-onset latency, total sleep time, and wake after sleep onset). SSM has been observed in insomnia (35–38), chronic fatigue syndrome (39), fibromyalgia (40–44), and irritable bowel syndrome (45).

Our results suggest that standard polysomnography may not reveal the full story of what the brain is doing during sleep in these disorders. The patient may be accurately perceiving this covert wakefulness, experiencing a state in which thought and memory processes similar to wakefulness occur in deep brain structures that go undetected by standard polysomnography. Indeed, there have been at least three studies that used standard polysomnography to look beyond sleep macrostructure (amount of time spent in each state, sleep-onset latency, etc.) toward sleep microstructure to find clues as to what characteristics influence SSM. Specifically, spectral properties in NREM sleep may distinguish between those with SSM and healthy controls (35, 40, 46–48). A more recent study has reported that N2 sleep in particular plays a critical role in self-reported sleep quality (49). Neuroimaging studies support this idea by showing that a subcortical network remains active during sleep in those with SSM (50, 51). These two studies found that arousal-promoting regions of the brain fail to show similar levels of deactivation during sleep in those with insomnia when compared with controls (50, 51).

In our data, nonsimultaneous states occurred throughout the night, and the amount of time spent in each state varied by region. Neuroimaging studies, like those mentioned above (50, 51) showing only 20- to 30-min time windows of differential sleep–waking states between areas during sleep, may still be reporting a portion of what normally occurs throughout the entire night. We found state pair combinations in which one region exhibited wake-like spectral properties lasting up to 26 min in length. Thus, there may be some baseline level of discrepancy in deactivation levels in even healthy control subjects. However, because these imaging studies compared SSM in patients with the brain activity of matched controls who we predict would also have some state discrepancies, perhaps overall there is more (e.g., 20 to 30 min more) sustained time spent in subcortical waking when the cortex is in NREM sleep in those with SSM. Indeed, we saw no epochs in which the subcortical hippocampus exhibited wakefulness while the cortex was simultaneously scored as being in N3 (*SI Appendix*, Table 1), whereas this is the combination found in SSM imaging studies. Future studies are required to determine the extent and types of nonsimultaneous states in healthy subjects and those with disordered sleep. The development of sleep-staging methods that facilitate both cortical and subcortical recordings will aid in this endeavor.

### Noninvasive State Determination of Deep Brain Structures.

Future work investigating the functions of sleep in humans and other animals will benefit from the analysis of recordings from deep brain structures when they are available. Magnetoencephalography can be considered for recording signals from deep brain regions noninvasively during sleep (52). Magnetoencephalography allows recordings of several hippocampal rhythms, which can be used for classifying sleep stages (53), and such recordings have been validated against iEEG (54). In cases where recordings from deep brain regions cannot be obtained in humans, it may be possible to discern subcortical states through the identification of unique features observed in cortical channels.

In our analyses of PSD profiles, we found that cortical signals during nonsimultaneous states exhibit frequency profiles intermediate between the power found in the assigned state of the cortex and the hippocampus. For example, in epochs where the cortex was scored as awake while the hippocampus was simultaneously scored as N2, the power in the 16 to 25 Hz range in the cortex was intermediate between that found in the same band during wakefulness (highest) and N2 sleep (lowest). In the case of epochs where the cortex was scored as REM while the hippocampus was simultaneously scored as N2, we again saw an intermediate spectral profile but in the slow-wave (0 to 2 Hz), theta (6 to 9 Hz), and spindle (10 to 15 Hz) bands. This intermediate PSD could be targeted to develop a biomarker for asynchronous states. Noninvasive determination of hippocampal states from scalp electrode signatures alone could refine data analysis and allow reexamination of prior studies. Reexamination of cortical signatures indicating subcortical states could reduce “noise” in the results or account for different results between studies due to previously unidentified differences in subcortical states.

## Conclusion

Taken together, these results reveal a need to approach sleep studies with a targeted focus on functionally relevant brain areas. Our revelation that whole sleep bouts can occur in humans independently in a subcortical brain area unregistered by surface cortical leads indicates that past study discrepancies could be reconciled by reviewing which brain area was assessed. Furthermore, future studies should take into account the understanding that regional sleep states between cortical and subcortical structures can and normally do occur beyond sleep onset, across the entire sleep period, and across phylogeny—rats, mice, birds, seagoing mammals, and now, also humans.

## Materials and Methods

### Subjects.

Scalp EEGs and iEEGs as well as elecrooculogram and electromyogram signals were recorded at the Department of Epileptology, University of Bonn from 14 patients diagnosed with pharmacologically intractable epilepsy. At the time of collection, informed consent and approval for the use of data for research purposes were obtained from all patients, and the study was approved by the Ethics Committee at the University of Bonn. Further details on data collection can be found in ref. 15.

### EEG Recordings.

Depth electrodes were referenced online to linked mastoids and recorded at a 1-kHz sampling rate. Details on data acquisition can be found in Staresina et al. (15) and Ngo et al. (14), and recordings are publicly available in the Open Science Framework repository (55). Further information on subject recordings used for analyses can be found in *SI Appendix*.

### Sleep Scoring.

Waking, non-REM stages N2 and N3, and REM sleep were scored in 30-s epochs by visual inspection using the Visbrain Sleep graphical user interface (56). Epochs containing large artifacts were visually marked and removed from the analyses. Sleep was scored for hippocampus and cortex independently, with the scorer blind to the scored state at the other site. Further details on sleep-staging criteria can be found in *SI Appendix*.

### Sleep Depth Characterization.

The standard sleep cycle transitions from waking to NREM stages 1 to 3 (N1 to N3), back briefly to N2, and to REM sleep, and then, the cycle begins again (57, 58). Traditional sleep hypnograms put wake at the top, indicating the lightest, most responsive state. Cortical EEG during REM sleep resembles the waking state, despite having arousal thresholds near to that of N3 sleep (59–62). However, we used the standard hypnogram depictions in accordance with many other characterizations in the literature.

We thus assigned each stage a “sleep depth score” on a scale of zero (awake) to three (deepest sleep). Specifically, each epoch marked as wake was scored as zero, REM was scored as one, N2 sleep was scored as two, and N3 sleep was scored as three. For the duration of the hypnogram, we took the hippocampal depth score minus the cortical depth score. When positive, the hippocampus was said to be in deeper sleep; when zero, the two regions were in a simultaneous sleep state, and when negative, the cortex was in deeper sleep. We then computed the fraction of the hypnogram that each participant spent with the hippocampus in deeper sleep, the cortex in deeper sleep, or the two sites in a simultaneous sleep state. To present overall findings, we took the mean value across subjects.

### Percentage of Time Spent in Each State.

Percentage of time spent in each state was calculated for each region separately by taking the total time in a respective state and dividing by the total duration of the hypnogram. Amount of time spent in each state was averaged across subjects and compared between the hippocampus and cortex using a two-tailed paired *t* test function from the Pingouin Python toolbox.

### State Pair Analysis.

Epochs in which the hippocampus and cortex were independently scored as being in the same or different sleep stages were defined as simultaneous or nonsimultaneous states, respectively. State pairs refer to the state assignments for both regions within an epoch.

The proportions of simultaneous and nonsimultaneous states were obtained to describe the amounts of time the hippocampus and cortex spend in each state separately or together. For each state (wake, N2, N3, REM), the total number of epochs where both brain regions were in the same state simultaneously and the total number of epochs each region was in that state were extracted. To obtain the proportion of simultaneity, the total number of simultaneous epochs was divided by the total number of epochs for the respective region.

### Bout Length.

A “bout” of one state in one region begins when that region enters that state and ends when that region moves to another state. We determined the duration of each bout for the hippocampus and the cortex by computing the number of 30-s epochs within the bout and dividing by two to yield a length in minutes. It should be noted that some bout lengths were underestimated, as epochs overwhelmed with artifacts were marked as “unscored” and thus, could interrupt continuous bouts. Statistical comparisons were completed in RStudio using the nlme library (63). The linear mixed effects regression model was fit using the maximum likelihood estimation with participant random intercepts and region fixed effects. The region fixed effects indicate the average difference between regions and were the primary focus of this analysis. Further details on statistical analyses can be found in *SI Appendix*.

Similarly, bout lengths were calculated as described above for bouts of each possible state pair between the hippocampus and cortex.

### Transition Diagrams.

To characterize the dynamics of how the brain transitions between sleep states, we computed the total number of epochs spent in each state pair. Each time point in the hypnogram was defined as either stable if the next time point was assigned the same state pair or a transition point if the state pair from the subsequent time point was different. For each pair of state pairs, {sp_i_,sp_j_}, we computed the total number of transitions from sp_i_ to sp_j_. We created transition diagrams using the digraph function in MATLAB (64), where the size of the node reflects the total number of epochs spent in each state pair and the shade of the directional arrow between them reflects the number of transitions. To present overall findings, we summed the number of epochs and transitions across all participants. To decrease visual clutter, arrows between states were omitted if there was only one such transition for individual participants or if there were five or fewer in the cross-subject sum. (Visual analysis comparing this method with taking the average of proportions indicated nearly identical results.)

### PSD Analysis.

To analyze the power spectral profiles in various state pair combinations, signals from each 30-s epoch were extracted from the hippocampal and cortical signals. PSDs were obtained using the YASA IRASA PSD function (65) in the frequency range of 0.25 to 30 Hz for each region and normalized between subjects by expressing them as a percentage of the total power across frequencies of all the same simultaneous state epochs within the recording. The total power was a single value calculated as the sum of the power across frequency bands for each state multiplied by the time spent in each respective state and calculated for the hippocampus and cortex separately. Mean power for each subject was obtained for each frequency bin, and power between state pairs was compared at corresponding frequency bins with a two-tailed paired *t* test. CIs for the 95th percentile are shown for each state pair. Further details on PSD analysis are in *SI Appendix*.

## Supplementary Material

Supplementary File

## Data Availability

Previously published data from ref. 55 were used for this work.
