# Supplementary material for: Recurrent Hippocampo-neocortical sleep-state divergence in humans
Source: Proc Natl Acad Sci U S A. 2022 Oct 24;119(44):e2123427119. doi: 10.1073/pnas.2123427119 (PMC9636919; doi:10.1073/pnas.2123427119)
Supplement: Supplementary File [file pnas.2123427119.sapp.pdf]

Supporting Information

Supplemental Methods

EEG Recordings

Data from fourteen subjects from this dataset were examined and data from eight subjects were used for the presented analyses. Four of 14 subjects were excluded due to having little sleep in their record in either hippocampus or neocortex. Because simultaneous states were used to normalize power between subjects for statistical comparisons (see Power Spectral Density Analysis), two other subjects were excluded due to the absence of any epochs of simultaneous REM sleep between the hippocampus and cortex, that is, all of their REM sleep was non-simultaneous.

Sleep scoring

Sleep was scored for hippocampus and cortex independently, with the scorer blind to the scored state at the other site. Criteria for scored states were according to Rechtschaffen & Kales 1968 and were as follows (1):

1. Wake (W) = low amplitude, mixed frequency or alpha activity (8-11 Hz).
2. NREM stage 2 (N2) = low amplitude and mixed frequency EEG with interruptions of sleep spindles (11-15 Hz) and slow oscillations or K complexes.
3. NREM stage 3 (N3) = high amplitude slow wave activity, with or without the presence of sleep spindles.
4. REM = low amplitude, mixed frequency activity with low power in the beta band (16-30 Hz).

Bout length

To compare bout lengths between the hippocampus and cortex we used a statistical test that would allow us to (a) account for differences in the number of observations because the hippocampus and cortex could have different numbers of bouts in each state and (b) account for repeated measures across participants due to a single subject contributing to multiple bouts from either the hippocampus or cortex. The linear mixed effects model allows us to account for within-person correlation that arises due to these repeated measurements. The linear mixed effects regression model was fit using maximum likelihood estimation with participant random intercepts and region-fixed effects. The region-fixed effects indicate the average difference between regions and were the primary focus of this analysis. This model takes the form of:

$$Y_i = b_0 + b_1 x_i + u_{0,i} + \varepsilon_i$$

where Y_i represents the bout lengths (within a state) for each person (i). b_0 is the fixed effect intercept, which represents the average bout length across individuals when

region=0 (i.e. the cortex). b_1 is the fixed effect slope for the region (x_i), which indicates the difference between the hippocampus (region=1) and cortex (region=0). $u_{0,i}$ is the random intercept, specified at the subject level, and is normally distributed [~N(0,σ^2)] representing the average deviation of a subject's individual intercept from the fixed effect intercept (b_0). Lastly ε_i is the term for the residual variance, is normally distributed [~N(0,σ^2)], and represents the difference between the subjects observed and model predicted score (i.e. the variance unexplained by the model).

Power Spectral Density Analysis

Power spectral density (PSD) profiles in various state pair combinations were extracted in 30s epochs from the hippocampal and cortical signals. Transition epochs, defined as the first and last epoch of contiguous state bouts, were removed as they could contain more than one state within the epoch. PSDs were obtained using the Welch periodogram method in the YASA IRASA (2) function where the Fast Fourier Transform was averaged over 4s windows of the signal with 50% overlap. Spectral values in the frequency range of 0.25-30 Hz for each region were normalized between subjects by expressing them as a percentage of the total power of all the same simultaneous state epochs and frequencies within the recording. The total power was a single value calculated as the sum of the power across frequency bands for each state multiplied by the time spent in each respective state. Total power was calculated for the hippocampus and cortex separately because each region showed consistently different amplitude signals based on the placement relative to the signal source and electrode resistances (all impedences were below 5 kΩ). For each frequency bin, we obtained the mean power for each subject and compared the power between state pair categories at each individual frequency using a two-tailed paired t-test. Confidence intervals (95th percentile) were obtained by randomly resampling the mean using 1000 Monte Carlo simulations (3).

Supplemental figure legends

Supplemental figure 1. (A) Hypnograms for each subject showing progression of sleep stages over the course of the overnight recording for the hippocampus (black) and cortex (gray). **(B)** The fraction of time one region is in deeper sleep than the other for each individual subject. Mean values across subjects can be found in **Figure 3a**.

Supplemental figure 2. Individual variability in state pair progressions across the night. A directed graph for each participant showing how state pairs transition from one

to another. Each node represents one possible state pair; the labels show the state pair and number of epochs that were scored as that pair. The size of the nodes reflects the relative prevalence of each state pair, and colors represent the states, with the inner color showing the state of the hippocampus (Wake: teal; N2: purple; N3: blue; REM: red) and the outer color showing the state of the cortex (very small nodes show only the hippocampal color). The directed edges of the graph show the total number of times a transition between two state pairs occurred. To simplify the graph, edges with only one transition are not shown. Note that the total number of transitions is much smaller than the total number of epochs, because long bouts of a single state pair account for many epochs but only one transition to/from that bout.

Supplemental table 1. Median bout lengths for all observed state pairs. State pairs are listed with hippocampal state listed first and cortical state listed second. The number of bouts, median and maximum lengths are provided along with the number of subjects (out of 8) that exhibited each state pair. The minimum length observed was 30s for each state pair and non-simultaneous states could last up to 36 minutes in length.

References

1. A. Rechtschaffen, A. Kales, A manual of standardized terminoloy, techniques, and scoring system for sleep stages of human subjects. *Arch. Gen. Psychiatry* **20**, 246–247 (1968).
2. R. Vallat, M. P. Walker, An open-source, high-performance tool for automated sleep staging. *eLife* **10**, e70092 (2021).
3. D. Ciliberti, F. Michon, F. Kloosterman, Real-time classification of experience-related ensemble spiking patterns for closed-loop applications. *eLife* **7**, e36275 (2018).

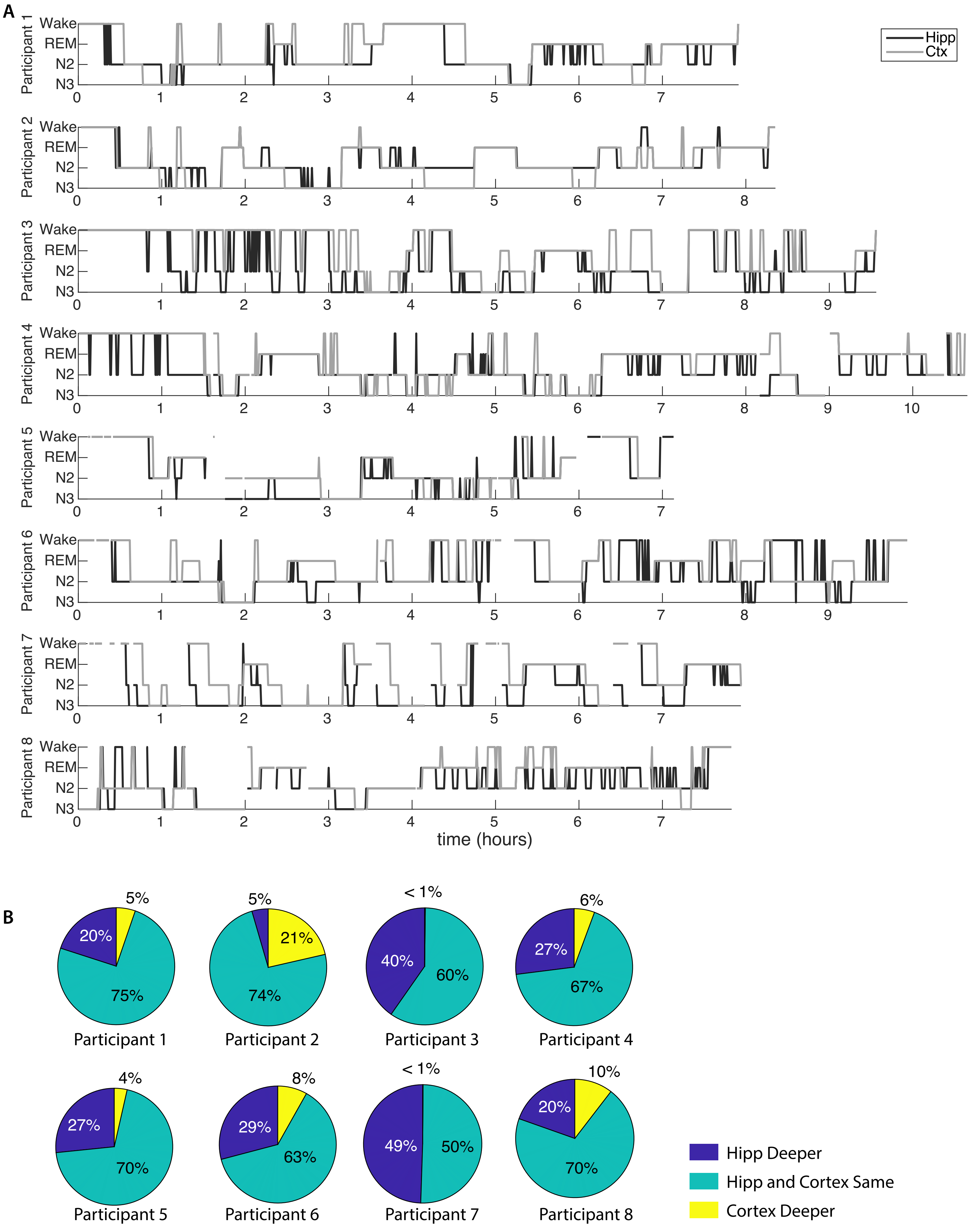

Supplemental figure 1.

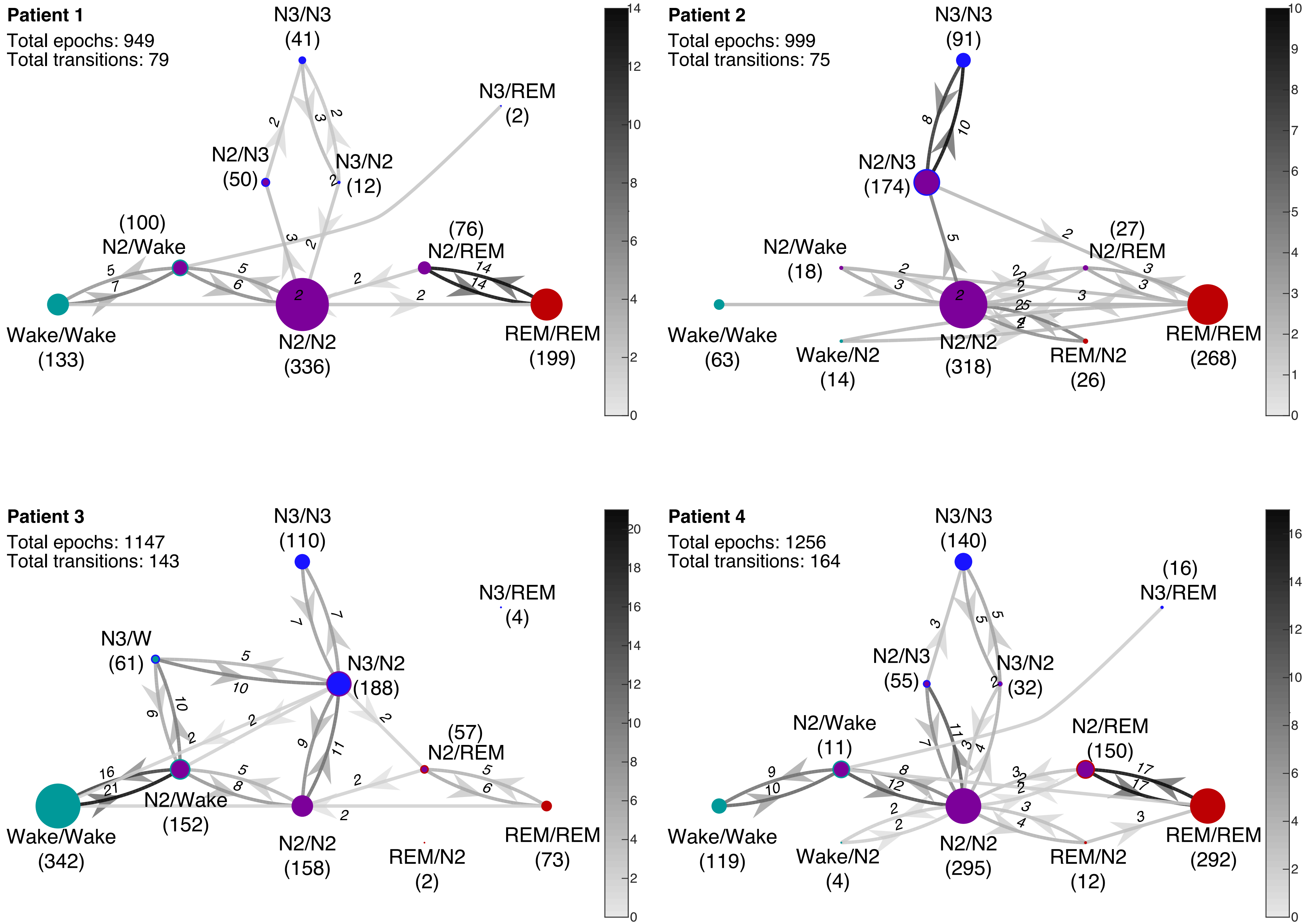

Supplemental figure 2.

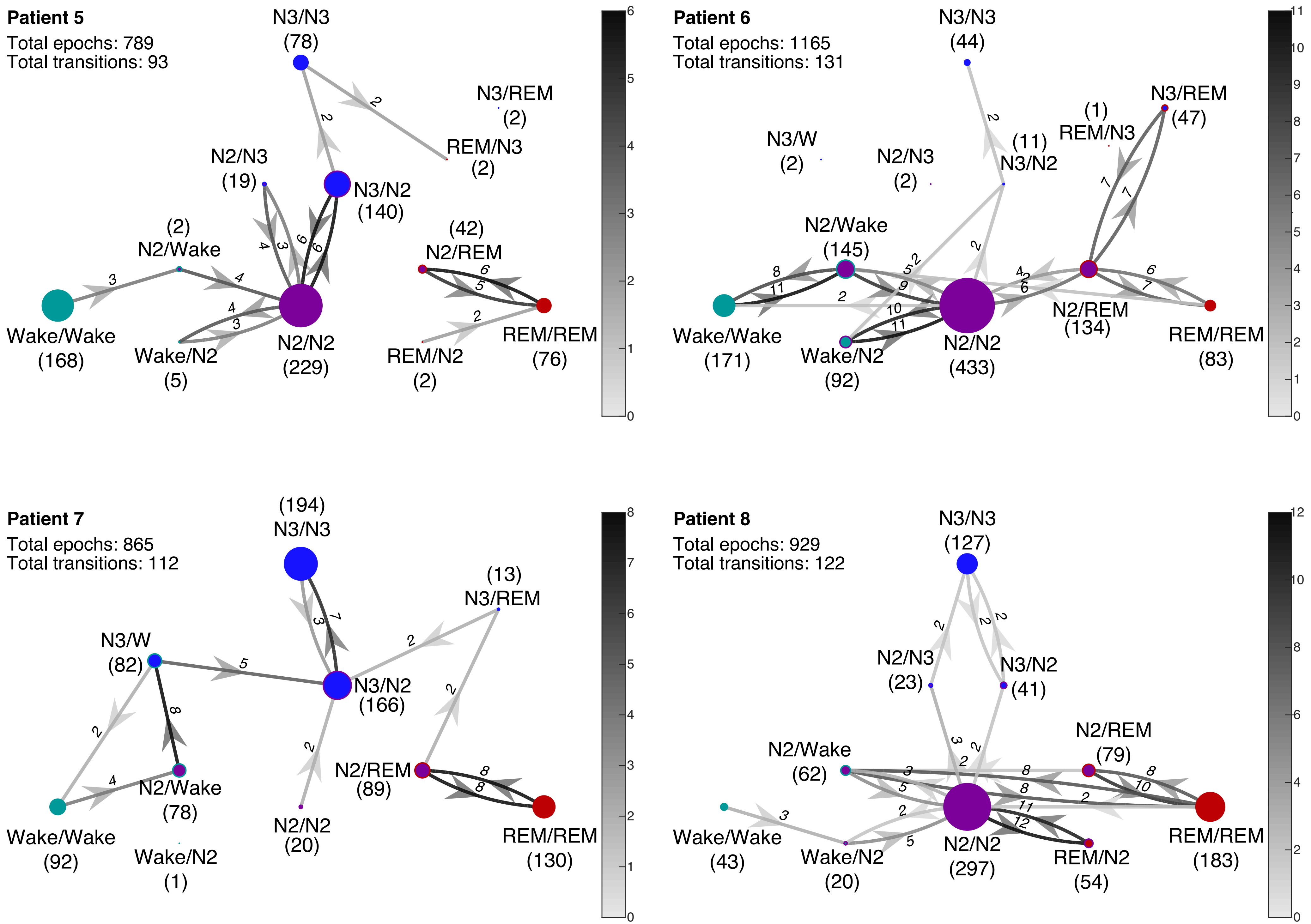

Supplemental figure 2 cont.

State Pair (Hipp/Ctx)	Count	Median (mins)	Max (mins)	Number of subjects
W/W	95	3.5	49	8
N2/N2	156	3.75	40	8
N3/N3	49	6.5	35.5	8
REM/REM	107	3.5	34	8
N2/W	124	2	26	8
N2/REM	98	2.25	16	8
N3/N2	75	2	31.5	7
N2/N3	38	1.5	36	5
W/N2	31	1	17	4
N3/REM	17	1.5	7.5	4
REM/N2	27	1	10	3
N3/W	27	2	9.5	2

Supplemental table 1.
